# Overexpression of *LtKNOX1* from *Lilium tsingtauense* in *Nicotiana benthamiana* affects the development of leaf morphology

**DOI:** 10.1080/15592324.2022.2031783

**Published:** 2022-02-10

**Authors:** Rui Zhou, Menglong Fan, Mei Zhao, Xinqiang Jiang, Qinghua Liu

**Affiliations:** a College of Landscape Architecture and Forestry, Qingdao Agricultural University, Qingdao, Shandong, China; b State Key Laboratory of Tree Genetics and Breeding, Research Institute of Subtropical Forestry, Chinese Academy of Forestry, Hangzhou, Zhejiang, China

**Keywords:** *Lilium tsingtauense*, KNOTTED-like homeobox, leaf morphology, *LtKNOX1*, leaf cell

## Abstract

Leaves are the main vegetative organs of the aboveground part of plants and play an important role in plant morphogenesis. *KNOTTED-LIKE HOMEOBOX* (*KNOX*) plays a crucial role in regulating leaf cell fate and maintaining leaf development. In this study, we analyzed *LtKNOX1* from *Lilium tsingtauense* and illustrated its function in transgenic plants. Tissue-specific expression analysis indicated that *LtKNOX1* was highly expressed in stems, young flower buds, and shoot apical meristems (SAMs). Ectopic overexpression of *LtKNOX1* in *Nicotiana benthamiana* suggested that transformants with mild phenotypes were characterized by foliar wrinkles and mildly curled leaves; transformants with intermediate phenotypes showed severely crimped blades and narrow leaf angles, and the most severe phenotypes lacked normal SAMs and leaves. Moreover, the expression levels of genes involved in the regulation of KNOX in transgenic plants were detected, including *ASYMMETRIC LEAVES1, PIN-FORMED 1, GA20-oxidase, CUP-SHAPED COTYLEDON 2, CLAVATA 1* and *WUSCHEL*(*WUS*), and the expression of other genes were down-regulated except *WUS*. This study contributes to our understanding of the *LtKNOX1* function.

## Introduction

Leaves are important vegetative organs and the main site of photosynthesis in the aboveground part of vascular plants. The initiation and formation of leaves are regulated by many genes, transcription factors, and miRNAs.^1^ The leaf primordium begins at the periphery of the shoot apical meristem (SAM) and develops into different leaf shapes and sizes.^2,3^ During this period, the transition from meristem to leaf requires correct cell proliferation and differentiation.^4,5^ Previous studies have reported that the coordinated expression of many important genes is required to regulate this physiological process, including *ASYMMETRIC LEAVES1* (*AS1*)/*ROUGH SHEATH2/PHANTASTICA, Homeodomain-leucine zipper III* (*HD-ZIP III*), and *PIN-FORMED 1* (*PIN1*).^6^ Among the gene families, KNOTTED-like homeobox (KNOX) transcription factor plays a central role in leaf morphogenesis by regulating leaf architecture and maintaining the meristem.^7,8^

KNOX, which belongs to the three amino acid loop extension (TALE) homeobox superfamily,^9,10^ is necessary for maintaining the function of meristem stem cells and establishing the initial boundaries between lateral organs in monocotyledons and dicotyledons.^2,11^ According to sequence similarity, the position of introns, and phylogenetic analysis of the homologous domain, *KNOX* can be divided into the class I *KNOX* and class Π *KNOX* subfamilies.^7,12^ *KNOTTED-1* (*KN1*) gene was the first homeobox gene isolated from the *Zea mays KN1* mutant, and the predicted gene product encoded a member of the homeodomain protein superfamily.^13,14^ The class I *KNOX* subfamily in *Arabidopsis thaliana* has four members including the *KN1*-like gene in *A. thaliana* (*KNAT1*)/*BREVIPEDICELLUS* (*BP), SHOOT MERISTEMLESS* (*STM), KNAT2*, and *KNAT6*, which could maintain the SAM and regulate the initiation of leaf primordia.^7,15^

*Lilium tsingtauense* (Liliaceae) is a rare wild lily mainly distributed in Qingdao, Shandong, China.^16^ Because of the destruction of its habitat in recent years, the number and distribution of *L. tsingtauense* have decreased, and it is now on the verge of extinction.^17^ The leaves of *L. tsingtauense* not only retain the feature of parallel veins in monocotyledons but have no sheath as in dicotyledons, such as *A. thaliana*. Therefore, the unique leaf characteristics of *L. tsingtauense* make it ideal for studying leaves. Although the function of *KNOX I* in other species has been confirmed, its function in lily remains unclear.

In this study, *LtKNOX1* was isolated and overexpressed in *Nicotiana benthamiana*. The phenotypes of the transformants revealed morphological changes in leaf shape, and the expression levels of the correlated genes. The results reveal the role of *LtKNOX1* from *L. tsingtauense* in the leaf development and provide genetic sources that could be useful for the molecular breeding of lily.

## Materials & methods

### Plant materials

*L. tsingtauense* was planted in the Qingdao Agricultural University experimental field (36.32° N, 120.39° E). The seeds of wild-type (WT) and transgenic *N. benthamiana* were sterilized with 75% ethanol and 2% sodium hypochlorite solution (NaClO), washed in sterile water and seeded on MS culture medium.^18^ The seeds were grown in a light incubator at 23 ± 2°C, 60–70% humidity, under a 16 h/8 h (light/dark) period, and 3,500 Lux light intensity. Developed SAMs, young leaves, mature leaves, stems, young flower buds, petals, and fruits of *L. tsingtauense* and mature leaves of *N. benthamiana* were collected in three replicates of each sampled tissue. Samples were then frozen in liquid nitrogen for 30 min and stored at – 80°C freezer until required.

### Isolating the LtKNOX1 fragment

The total RNA of 6-week-old *L. tsingtauense* leaves was extracted using the RNAprep Pure Plant Plus Kit (Polysaccharides & Polyphenolics-rich) (TIANGEN, Beijing, China) and reverse-transcribed with HiScript ® III All-in-one RT SuperMix Perfect for the quantitative polymerase chain reaction (qPCR; Vazyme, Nanjing, China). The *LtKNOX1* sequence was separated from the *L. tsingtauense* transcriptome (Accession number: PRJNA497597) (Supplemental Table S1). The *LtKNOX1* forward and reverse primers were designed with Primer Premier version 5.0 (PREMIER Biosoft International, San Francisco, CA, USA) based on the *L. tsingtauense* transcriptome (http://www.ncbi.nlm.nih.gov/Traces/sra) (Supplemental Table S2). The target fragments were purified and recovered using the FastPure Gel DNA Extraction Mini Kit (Vazyme, Nanjing, China).

### LtKNOX1 bioinformatics and phylogenetic analyses

The open reading frame (ORF) of the separated *LtKONX1* sequence was analyzed using the NCBI ORF Finder (https://www.ncbi.nlm.nih.gov/orffinder/). The LtKNOX1 conserved domain was predicted using Smart (http://smart.embl-heidelberg.de/), and the basic physicochemical properties of the LtKNOX1 protein were analyzed using Expasy ProtParam online analysis software (http://web.expasy.org/protparam/). The sequencing results were compared and analyzed using DNAMAN version 8.0 (Lynnon Biosoft, Quebec, Canada). The amino acid sequences of the KNOX family members used in this study were downloaded from the NCBI (https://www.ncbi.nlm.nih.gov/), and the phylogenetic tree was constructed using the maximum-likelihood (ML) method with Jones-Taylor-Thornton (JTT) model in MEGA-X,^19^ the accession numbers of the sequences used in the phylogenetic tree were provided in the Supplemental Table S3.

### Subcellular localization

The coding sequence (CDS) of *LtKNOX1* minus the stop codon was inserted upstream of the green fluorescent protein (GFP) reporter gene to construct the pSUPER1300-LtKNOX1-GFP fusion vector and pSUPER1300-GFP (empty plasmid as control).^20^ The forward and reverse primers are listed in Supplemental Table S2. We used pSUPER1300-GFP and pSUPER1300-LtKNOX1-GFP *N. benthamiana* during the vegetative growth stage. The leaves of the WT and transformants were collected, cut into fresh slices, and the GFP fluorescence signals were observed with a Leica DM2500 fluorescence microscope.^21^

### Acquisition of transformants

pSUPER1300 with the CaMV35S promoter was used to construct pSUPER1300*-LtKONX1*, followed by *Agrobacterium tumefaciens*-mediated transformation.^22^ The transformants were screened on MS medium containing 30 mg/L hygromycin. When the transformants produced 4–6 true leaves, DNA was extracted using the CTAB method,^23^ and the positive transformants were detected and identified by PCR (Supplemental Figure S1). We selected three lines according to the phenotypes of transgenic lines and the expression of *LtKNOX1* among different lineages. Overexpressing line 3 (OE#3) used the T1 generation because it could not blossom and bear normal fruit, overexpressing line 1 (OE#1) and overexpressing line 2 (OE#2) used the T2 generation. The primers used for constructing the overexpression vector are listed in Supplemental Table S2.

### Morphological analysis of the transformants

The growth differences between the WT and transformant plants were determined to characterize changes in leaf shape. Leaf width, leaf length, the leaf shape index (LSI), and the curvature index (CI)^24^ of each plant that germinated after 30, 50, and 70 days was measured with Vernier calipers. Plant traits, including leaf angle (LA), petiole length, petiole width, internode length, and plant height^25^ were measured 70 days after germination (reproductive growth stage). Different letters marked on each column represent significant differences when analyzed by one-way ANOVA and a multiple comparison using Least-significant difference(LSD) at *P* < 0.05.

### Microstructural analysis

Mature leaves (totally curled) of WT and transformant plants were brushed with transparent nail polish to form a thin coating on the upper and lower epidermis of the leaves and held at room temperature. After drying, the film formed by the nail polish was removed and placed on a temporary slide.^26^ The adaxial and abaxial cells of the leaves were observed using a Leica DM2500 fluorescence microscope. Image J^27^ was used to calculate the number and size of the cells.

### Quantitative real-time polymerase chain reaction (qRT-PCR) analysis

The six types of *L. tsingtauense* tissues mentioned above and the leaves of 8-week-old transgenic *N. benthamiana* plants were used for qRT-PCR. The thermal cycling parameters for qRT-PCR were: pre-denaturation at 95°C for 20s, followed by 40 cycles of 95°C for 10s, 56°C for 30s, and 72°C for 30s. *LtGAPDH* (*glyceraldehyde-3-phosphate dehydrogenase*) and *NbGAPDH* were used as the control housekeeping genes in the *L. tsingtauense* and transgenic *N. benthamiana* lines respectively.^28^ The *N. benthamiana* genes for qRT-PCR were obtained from the *N. benthamiana* genomic database (https://btiscience.org/). The transcript levels were qualified using the 2^−∆∆CT^ method.^29^ Each reaction was repeated three times. All primers used in the qRT-PCR are listed in Supplemental Table S2.

## Results

### Identification and phylogenetic analyses of LtKNOX1

**Figure 1.**
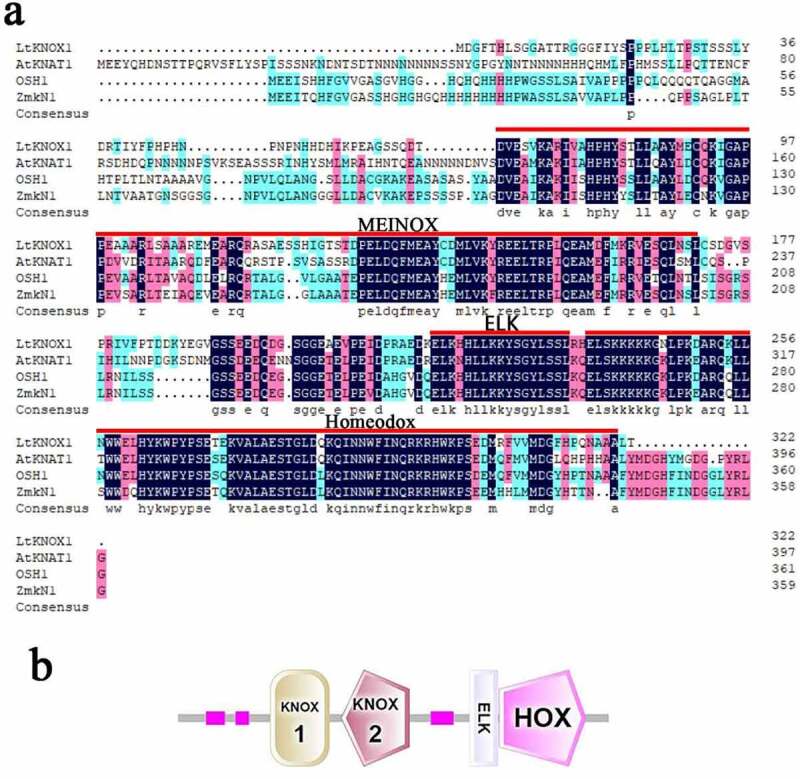
*LtKNOX1* multiple sequence alignment. (a) The amino acid sequence alignment of LtKNOX1 and class I KNOX members of other species (*Arabidopsis thaliana, Zea mays, Oryza sativa*). The red solid line corresponds to the region of each conserved domain (MEINOX, ELK, Homeobox). (b) The conserved domains of LtKNOX1 predicted by Smart. The small pink boxes are regions of low compositional complexity.

The *LtKNOX1* sequence was selected from the *L. tsingtauense* transcriptome (Genbank Accession number: PRJNA497597).^30^ The *LtKNOX1* gene (Genbank Accession number: OK554547) was annotated in the Gene Ontology (GO) (Biological Process: cell fate specification, GO:0001708), Pfam (PF03791), Non-Redundant Protein Sequence Database (NR) (gi|383212085|dbj|BAM08929.1|) and Swiss-Prot (tr|O04135|KNAP2_MALDO) databases. The complete CDS of *LtKNOX1* was 969 bp and encoded 322 amino acids (Figure 1a and Supplemental Table S1). The predicted molecular weight of LtKNOX1 was 36,328.6 kDa, the instability index was 46.92, and the grand average of hydropathicity was – 0.815. These results indicate that LtKNOX1 was a stable and hydrophilic protein (Table 1). LtKNOX1 contained the highly conserved homeobox family domains of MEINOX (KNOX1, KNOX2), ELK, and Homeobox (Figure 1b).

**Table 1.** The physicochemical properties of LtKNOX1 in *L. tsingtauense.*

Gene name	CDS (bp)	Amino Acids	Molecular weight (KDa)	Theoretical pI	Instability index	Aliphatic index	Grand average of hydropathicity
*LtKNOX1*	969	322	36328.60	6.06	46.92	62.48	−0.815

**Figure 2.**
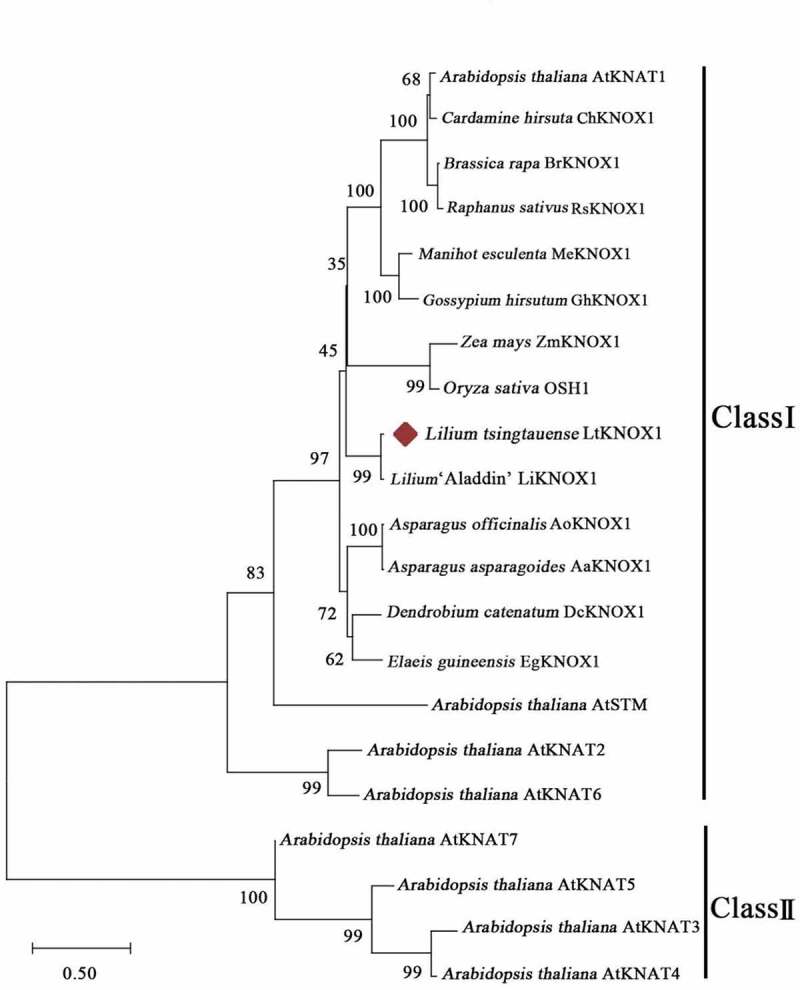
Phylogenetic analysis of LtKNOX1. Phylogenetic tree of the protein sequences of 17 genes encoding class I KNOX and 4 genes encoding class Π KNOX from 14 angiosperms. The red solid rhombus indicates the target amino acid sequence. The species are: *Arabidopsis thaliana; Cardamine hirsuta; Brassica rapa; Raphanus sativus; Manihot esculenta; Gossypium hirsutum; Zea mays; Oryza sativa; Lilium tsingtauense; Lilium* ‘Aladdin’ (*Lilium longiflorum × Lilium asiatic); Asparagus officinalis; Asparagus asparagoides; Dendrobium catenatum; Elaeis guineensis.*

The phylogenetic tree showed that class I KNOX in monocotyledons and eudicotyledons were clearly separated. The function of the class I KNOX subfamily may differ between monocotyledons and dicotyledons (Figure 1c). LtKNOX1 was evolutionarily closely related to KNOX1 of *Lilium* ‘Aladdin’ (*Lilium longiflorum × Lilium asiatic*) (LiKNOX1)^31^ in these species (Figure 2). The Accession numbers of the sequences used in the phylogenetic tree are listed in Supplemental Table S3.

### Tissue-specific expression and subcellular localization analysis

**Figure 3.**
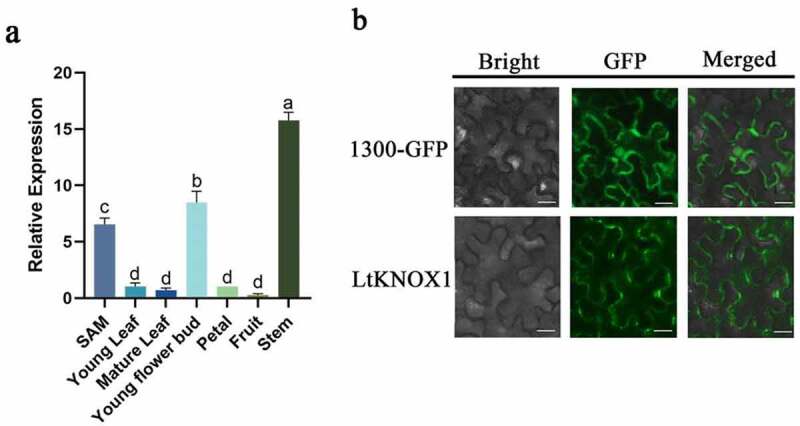
The expressed analyses of LtKNOX1. (a) The relative expression of *LtKNOX1* in different tissues of *L. tsingtauense*. Data indicate means ± SD (n = 3 replications for each validation), and the expression of *LtKNOX1* in the petal was set to 1. (b) Subcellular localization of LtKNOX1. Observation of GFP fluorescence in leaf of pSUPER1300-GFP (control) and pSUPER1300-LtKNOX1-GFP *N. benthamiana*. Merged: GFP and bright-field merged images. Scale bars = 100 μm.

The qRT-PCR analysis of six *L. tsingtauense* organs was performed. The expression level of *LtKNOX1* was higher in stems, young flower buds, and SAMs, but significantly lower in leaves (young and mature leaves), petals and fruit tissues (Figure 3a and Supplemental Table S4). These results showed that LtKNOX1 expression had stage and tissue specificity.

The subcellular localization of LtKNOX1 with a GFP vector in transgenic *N. benthamiana* leaves revealed that pSUPER1300-LtKNOX1-GFP was located in the cell membrane of leaf, and pSUPER1300-GFP of the control was located in the cell membrane and nucleus (Figure 3b).

### Leaf shape index and curvature of the transformants

**Figure 4.**
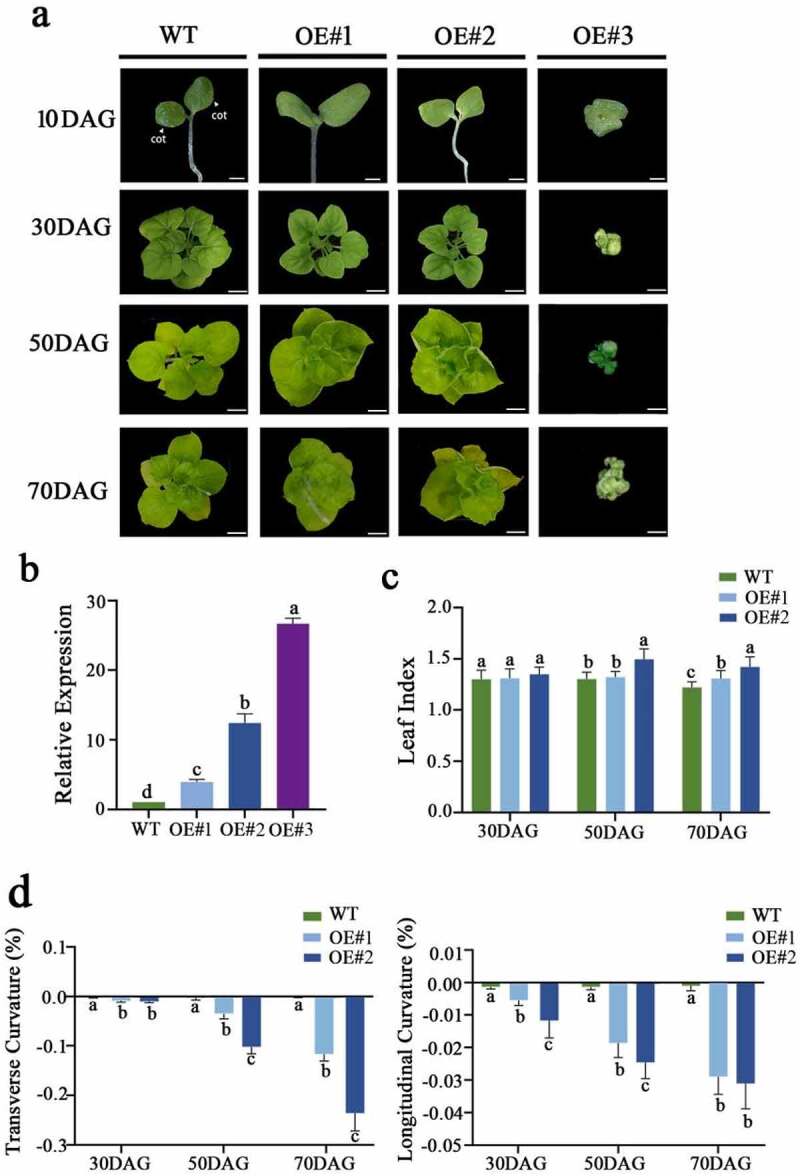
Phenotypes and the relative expression of *LtKNOX1* in the transformants. (a) Observation of the postembryonic development of WT and transformants. The white arrow indicates cotyledons. (b) The relative expression of LtKNOX1 in transformants. The expression of genes in the WT was set to 1, and data indicate mean ± SD (n = 3 replications for each validation). (c) Leaf shape index of WT, OE#1, and OE#2 in different developmental stages. (d) The longitudinal curvature index (LC) and transverse curvature index (TC) of different stages of leaves of WT, OE#1, and OE#2. DAG: days after germination. Data indicate mean ± SD (n = 10 replications for each validation). Scale bars = 500 μm (a, 10 DAG); Scale bars = 1 cm (a, 30-70 DAG).

The leaf phenotypes of the transformants from the three lines ranged from mild to severe: OE#1, OE#2, and OE#3 (Figure 4a). The *LtKNOX1* expression level was highest in OE#3 (Figure 4b and Supplemental Table S4). Therefore, we speculated that the different degrees of phenotypic variation in the transgenic plants may be related to the LtKNOX1 expression. Because OE#3 did not produce normal leaves, we measured the leaf characters of OE#1 and OE#2.

To characterize changes in leaf shape during leaf curling, we measured the width and length of leaves before and after flattening, and the LSI after flattening during different growth periods. The transformants leaves were narrower than the WT after flattening during the middle vegetative stage and reproductive stage (Table 2), but there was no difference in leaf length (Table 3). Therefore, the LSI of transformants was lower than that of the WT, and the leaf shape of WT was more round than that of the transformants (Figure 4c). Especially 70 days after germination (DAG), the width of OE#1 and OE#2 mature leaves of increased by 11.63% and 23.34% after flattening, respectively, which was significantly higher than that of the WT, and the variation in the leaf length was low (Tables 2 and 3).

**Table 2.** The leaf width of WT and transformants before and after flattening during different growth stages

Lines	Leaf Width (cm)								
	30 DAG		50 DAG				70 DAG		
	Before flattening	After flattening	Increase rate	Before flattening	After flattening	Increase rate	Before flattening	After flattening	Increase rate
WT	1.78 ± 0.08	1.78 ± 0.08	0.26%	2.78 ± 0.13	2.79 ± 0.13	0.27%	3.23 ± 0.05	3.23 ± 0.05	0.15%
OE#1	1.72 ± 0.10	1.73 ± 0.10	0.93%	2.69 ± 0.10	2.78 ± 0.10	3.43%	2.77 ± 0.14	3.10 ± 0.12	11.63%
OE#2	1.68 ± 0.05	1.70 ± 0.05	1.02%	2.24 ± 0.12	2.47 ± 0.15	10.27%	2.38 ± 0.09	2.93 ± 0.20	23.34%

The initial stage measurement was carried out when the leaf was not crimped, at 30 days after germination (DAG). At 50 DAG, the leaf width was determined when the rolled leaves were formed. At 70 DAG, the width of mature leaves was determined when the leaves were completely curled. Data indicate mean ± SD (n = 10). The increase in leaf width (%) was measured by comparing the values before and after flattening (× 100).

**Table 3.** The leaf length of WT and transformants before and after flattening during different growth stages

Lines	Leaf Length (cm)								
	30 DAG			50 DAG			70 DAG		
	Before flattening	After flattening	Increase rate	Before flattening	After flattening	Increase rate	Before flattening	After flattening	Increase rate
WT	2.31 ± 0.13	2.32 ± 0.13	0.12%	3.62 ± 0.07	3.63 ± 0.07	0.13%	3.93 ± 0.17	3.95 ± 0.17	0.41%
OE#1	2.25 ± 0.06	2.26 ± 0.06	0.42%	3.66 ± 0.07	3.67 ± 0.07	0.45%	3.98 ± 0.14	4.04 ± 0.13	1.52%
OE#2	2.27 ± 0.12	2.30 ± 0.11	1.08%	3.66 ± 0.08	3.68 ± 0.09	0.72%	4.15 ± 0.20	4.17 ± 0.20	0.47%

The initial stage measurement was carried out when the leaf was not crimped, at 30 days after germination (DAG). At 50 DAG, the leaf length was determined when the rolled leaves were formed. At 70 DAG, the length of mature leaves was determined when the leaves were completely curled. Data indicate mean ± SD (n = 10). The increase in leaf length (%) was measured by comparing the values before and after flattening (× 100).

To accurately quantify the degree of blade crimping, we measured the CI of the transformants and the WT leaves. The longitudinal curvature index (LC) and transverse curvature index (TC) of OE#1 and OE#2 leaves were less than zero at different stages. There was little difference between the leaf curvature of the transformants and the WT at 30 (DAG). TC became significantly lower than LC as the plants developed (Figure 4d and Supplemental Table S5), indicating that the degree of leaves crimping gradually increased with the maturity of the leaves, and leaf crimping mainly occurred on the horizontal axis. These results indicate that overexpression of *LtKNOX1* affected the lateral growth of leaves.

**Figure 5.**
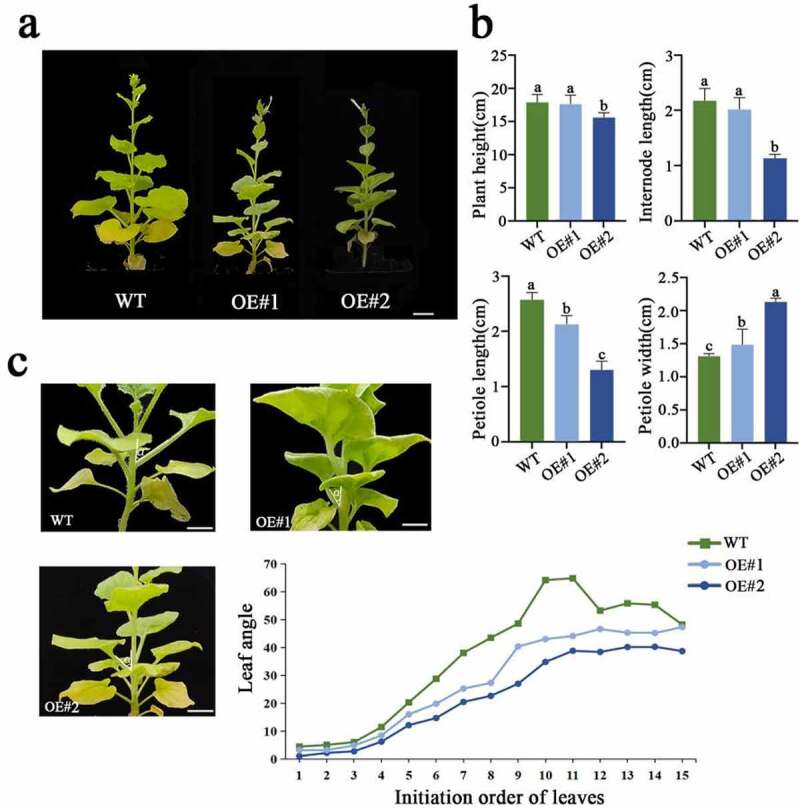
Morphological phenotypes and leaf angle variations in the transformants. (a) The overall phenotypes of 70 days after germination (DAG) transformants. (b) Phenotypic statistics of mature plants in the reproductive growth stage. (c) The leaf angle between the adjacent leaves and stems of mature plants. Data indicate mean ± SD (n = 10 replications for each validation). Scale bars = 1 cm.

In addition, we found that the petioles of transformants was significantly shorter than those of the WT, and that the petiole width of the transformants was significantly greater compared to the WT (Figure 5a,b and Supplemental Table S6). The variation in these traits, along with leaf angle and leaf curling, made the transgenic plants more compact. The leaf inclination angle of transformants was smaller than that of the WT, and the leaf inclination angle of OE#2 with severe leaf curling was the smallest among the transformants (Figure 5c). The leaf angle of the transgenic plant was smaller than that of the WT, particularly the middle leaf (from the fifth leaf to the eleventh leaf) (Figure 5c).

### Leaf deficiency and ectopic buds of OE#3

The OE#3 transformants did not form normal apical meristems (Figure 4a, Figure S2). The green leafy organs of OE#3 were extremely small, irregular, and thick. The growth of these green leafy organs was slow, and the leaf veins were absent in the gradually expanding leaves. Radial symmetry appeared in some leaves near the SAM when the number of these organs increased and the plants increased in height. With the development of amorphous green leaf organs, these organs no longer have the characteristics of common leaves. We often observed ectopic buds in the axils of these coral-shaped leaves, but they did not develop into normal stems or leaves.

### Microstructure of the adaxial and abaxial sides of the leaves

**Figure 6.**
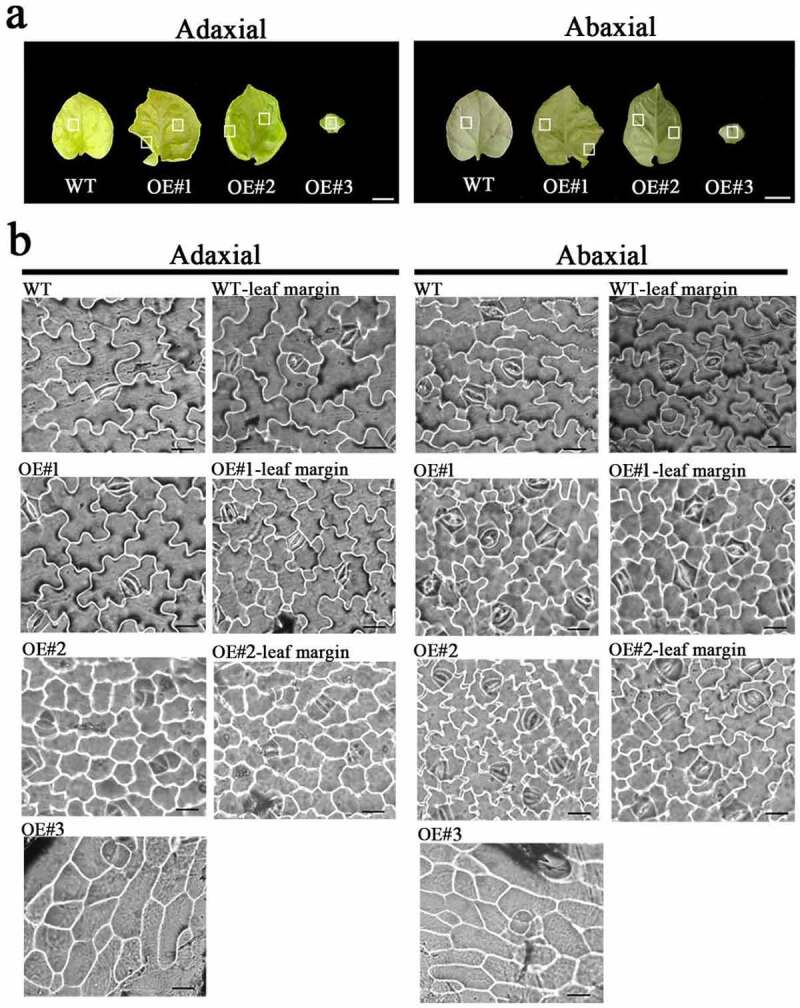
Observations of the ad/abaxial sides of the transformants. (a) The mature leaves of transformants and WT. The white solid rectangles indicate the region for microscopic observation and cell quantification. (b) Micro-structure of ad/abaxial sides of transformants and the WT. The images were taken with a fluorescence microscope; and the position on the adaxial and abaxial sides was exactly the same. Scale bars = 1cm (a); Scale bars = 20 μm (b).

To illustrate the variations in leaf morphology at the cellular level, we observed the micro-structure of the adaxial and abaxial surfaces of the leaves of the WT and the transgenic lines (OE#1, OE#2 and OE#3) (Figure 6a). The abaxial surfaces of the OE#1 were irregularly shaped with shrunken cells, whereas the adaxial surface cells look like the WT (Figure 6b). The cell morphology of OE#2 could not be distinguished between the adaxial and abaxial surfaces (Figure 6b). The abaxial cells in the middle part of the leaves of OE#2 were irregularly arranged (Figure 6b). The OE#3 cells displayed different shapes, did not produce structures similar to adaxial and abaxial cells, were arranged loosely and irregularly, and lost their differentiating function into leaf cells.

**Figure 7.**
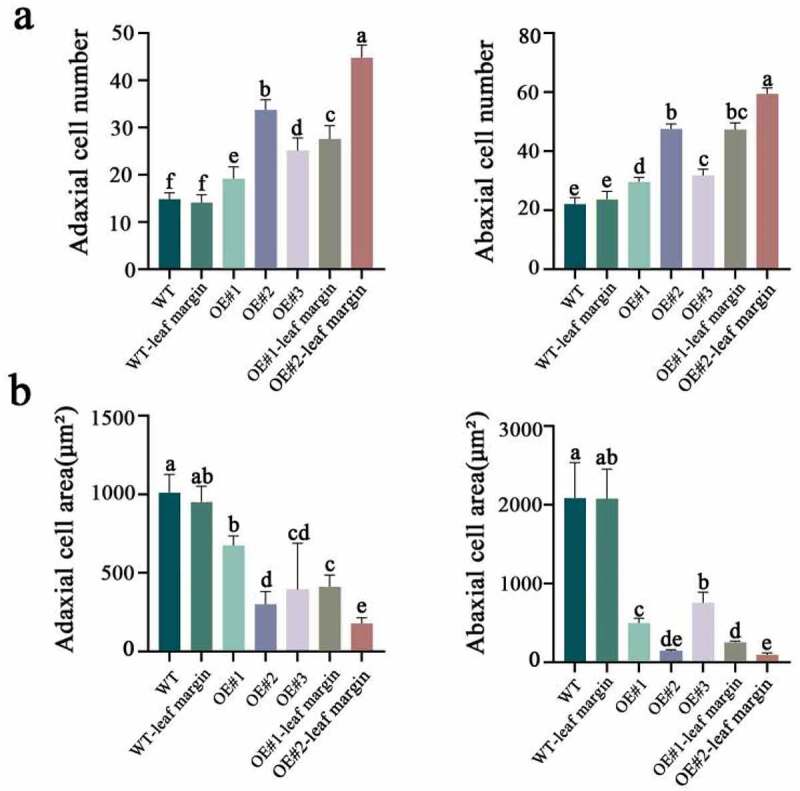
The cell number and area of the ad/abaxial sides of the transformants. (a) The cell number of ad/abaxial sides. (b)The cell area of ad/abaxial sides. The number and area of cells were randomly selected from ten visual fields with the same magnification. Data indicate mean ± SD (n = 10 replications for each validation).

In addition, we quantified the cells on the adaxial and abaxial surfaces of the above observed microstructural area. Significantly more cells were observed in the transformants than in the WT (Figure 7a and Supplemental Table S7). Furthermore, the area of the periclinal external wall decreased significantly, particularly in the leaf margins of OE#1 and OE#2 (Figure 7b), indicating that the cells were under severely suppressed expansion particularly in the curled part of the leaf margin. These results indicate that heterologous overexpression of *LtKNOX1* changed the leaf cell fates.

### Expression of related genes in transgenic plants

**Figure 8.**
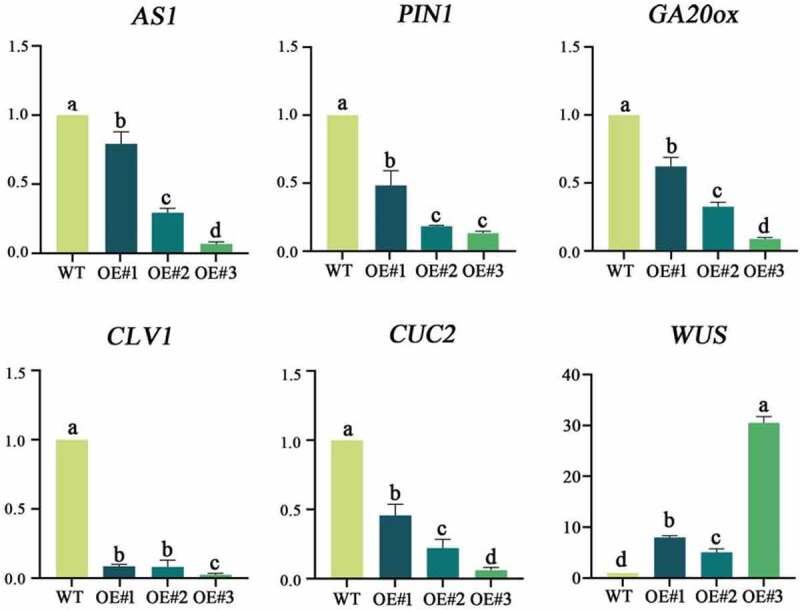
The qRT-PCR for related genes regulated by the KNOX1 in transformants curl leaves. Data indicate mean ± SD (n = 3 replications for each validation), and the expression of genes in the WT was set to 1.

Based on the phenotypes of the *LtKNOX1*-overexpressing transformants, we analyzed the regulatory role in *LtKNOX1* and other related genes of transgenic plants using the WT as a control. We examined six genesincluding *AS1, PIN-FORMED 1* (*PIN1), GA20-oxidase* (*GA20ox), CUP-SHAPED COTYLEDON 2* (*CUC2), CLAVATA 1* (*CLV1*) and *WUSCHEL* (*WUS*) in transformants. The expression level of *WUS* was up-regulated in the three transgenic lines, whereas the expression of the other genes was down-regulated in the transformants. The expression levels of *AS1, PIN1, GA20ox*, and *CUC2* in transformants were significantly different among the transgenic lines (Figure 8 and Supplemental Table S4).

## Discussion

In this study, LtKNOX1 was localized on cell membrane. Although some studies have reported KNOX I was localized on nucleus, it was also found that KNOX was localized on the cell membrane except the nucleus.^32–37^ Therefore, the subcellular localization pattern is different in species. In addition, the subcellular localization of the LtKNOX1 protein may be affected by intercellular transport.

In this study, we demonstrated that ectopic overexpression of *LtKNOX1* affected leaf shape. This finding is similar to the expression of most class I *KNOX1*-like genes observed in other species.^38^ The expression of class I *KNOX* is limited to the SAM in single leaf plants, and then down-regulated in the leaf primordium.^2,7^ The decrease in leaf size may be caused by inhibition of the gibberellic acid (GA) biosynthetic pathway by the *KNOX* gene.^7,9,39^ Interestingly, the transformants with mild and moderate phenotypes displayed smaller leaf angle, indicating that overexpression of *KNOX1* affected the lateral growth of leaves. In *O. sativa*, OsOFP6 regulates leaf angle and interacts with the class I KNOX protein OSH15, which enhances the transcriptional activity of OSH15. This interaction results in a change in leaf angle.^25^ In addition, the petiole of transgenic plants became shorter and thicker. A previous study reported that *A. thaliana* HDA6, which is a part of the AS1 repression complex, may regulate *KNOX* expression resulting in the short leaf petiole phenotype.^40^ Therefore, the variation in the petiole may be related to the interaction between *AS1* and *KNOX1*. The transformants with the most severe phenotype developed fused green leaf organs and ectopic buds in the axils of the leaves. Similar phenotypic abnormalities were observed in overexpressing *O. sativa OSH1, A. thaliana KNAT1*, and *Populus tremula× tremuloides PttKN1*.^41–43^

The proliferation and differentiation of cells directly affect the leaf, and this is a key step driving the development of leaves.^44^ The class I KNOX is the key transcription factor supporting the SAM maintenance including the indeterminate cell fates.^45^ The ectopic overexpression of class I *KNOX* genes leads to variations in the transition from the cell proliferative to the expansion/differentiation phases.^11^ In this study, the histological observations revealed a variation in the proliferation and differentiation of ad/abaxial side cells. In *Z. mays, Rough sheath1* (*RS1*, class I *KNOX* gene) alters cell fate and causes unregulated cell division and expansion of leaves.^46^ Overexpression of *N. tabacum* class I *KNOTTED*-like homeobox (*NTH*) leads to abnormal leaf morphology. The degree of leaf bending of in the *NTH* transgene was slightly different, which was related to the level of transgenic expression.^47^ Abnormal cell proliferation in *O. sativa opb* mutants may be caused by ectopic class I *KNOX* gene expression.^48^ Ectopic expression of TKn4 in tomato leads to shrinking leaf, which inhibits differentiation of meristems and vasculature.^49^ The overexpression of the potato *homeobox 15* (*POTH15*) gene causes changes in the arrangement of leaf and stem cells.^50^

As a key transcription factor in plant development, the class I *KNOX* gene maintains the function of SAM by regulating the expression of other key genes. The leaves of transgenic plants are curled and lose apical dominance in severe cases, which is similar to the *A. thaliana as1* mutant.^51,52^ qRT-PCR detection of transgenic plants showed that the expression of *AS1* was decreased, especially in OE#2, suggesting that the *LtKNOX1* gene plays a role in inhibiting *AS1* gene. Previous studies have reported that AS1 and AS2 form a protein complex that inhibits the expression of *KNAT1/BP* and *KNAT2* resulting in regular leaf morphology. *PIN1* also decreased in the transformants. There is an antagonism between KNOX and auxin. AS1 and AS2 with auxin may synergistically inhibit the expression of *KNOX*. The interaction among auxin, AS1 and class I KNOX may directly affect leaf germination and leaf morphology.^53^ However, whether class I KNOX protein interferes with the auxin flow in stem tips and leaf primordia and results in a leaf phenotype similar to that of *as1* mutant remains unclear, but evidence indicates that class I KNOX regulates the synthesis and decomposition of GA. The suppression of *GA20ox* in transformants leaves was validated, which was consistent with previous results. The overexpression of the class I *KNOX* gene *NTH15* in *N. tabacum* inhibits the expression of *Ntc12* (*GA20-oxidase*).^54^ KNOX directly inhibits the synthesis of GA, which contributes to establish the boundary between SAM and the primary leaf primordia.^7,55–57^ KNOX1 decreases the leaf area in *A. thaliana as1* and *as2* mutants by inhibiting the GA pathway, and this negative pathway may be conserved in species with different genetic backgrounds.^58,59^ Correct formation of the SAM is the premise for the development of lateral organs. Ectopic expression of class I *KNOTTED-like homeobox* genes leads to the formation of the ectopic meristem.^60–62^ In the SAM, WUS and STM interact directly and bind the *CLV3* promoter to activate its expression, thus regulating stem cell activity in the SAM. The *WUS* expression was higher and the *CLV1* expression was lower in OE#2, which had a severe SAM-deficient phenotype, compared to the WT.

## Conclusion

In this study, we isolated and overexpressed the *LtKNOX1* gene in *N. benthamiana*. Morphological and histological analyses revealed that the leaf shape of the transformants was destroyed. The qRT-PCR analysis showed that *LtKNOX1* regulated the expression of several genes involved in the formation of the SAM and the development of leaves. These results indicate that *LtKNOX1* affects the development of leaf morphology in *N. benthamiana*. Therefore, the results provide valuable insight into the function of *LtKNOX1* which could be used to enhance the *Lilium* spp. breeding in the future.

## Supplementary Material

Supplemental Material

## References

[1] Cammarata J, Roeder AHK. Development: cell polarity is coordinated over an entire plant leaf. Curr Biol. 2018;28(16):R884–12. doi:10.1016/j.cub.2018.07.007. 30130511

[2] Scofield S, Murray JA. KNOX gene function in plant stem cell niches. Plant Mol Biol. 2006;60:929–946. doi:10.1007/s11103-005-4478-y. 16724262

[3] Somssich M, Je BI, Simon R, Jackson D. CLAVATA-WUSCHEL signaling in the shoot meristem. Development. 2016;143(18):3238–3248. doi:10.1242/dev.133645. 27624829

[4] Du F, Guan C, Jiao Y. Molecular mechanisms of leaf morphogenesis. Mol Plant. 2018;11(9):1117–1134. doi:10.1016/j.molp.2018.06.006. 29960106

[5] Nikolov LA, Runions A, Das Gupta M, Tsiantis M. Leaf development and evolution. Curr Top Dev Biol. 2019;131:109–139. doi:10.1016/bs.ctdb.2018.11.006. 30612614

[6] Chai L, He J, Gao Z, Zhang W, Feng B, Han H, Xu C, Zeng Z, Wu L. Advances in plant leaf development and morphogenesis research. Seed. 2018;37:46–48.

[7] Hay A, Tsiantis M. A KNOX family TALE. Curr Opin Plant Biol. 2009;12(5):593–598. doi:10.1016/j.pbi.2009.06.006. 19632142

[8] Scofield S, Dewitte W, Murray JA. STM sustains stem cell function in the Arabidopsis shoot apical meristem and controls KNOX gene expression independently of the transcriptional repressor AS1. Plant Signal Behav. 2014;9:e28934. doi:10.4161/psb.28934. 24776954 PMC4091562

[9] Cruz R, Melo-de-pinna GFA, Vasco A, Prado J, Ambrose BA. Class I KNOX is related to determinacy during the leaf development of the Fern Mickelia scandens (Dryopteridaceae). Int J Mol Sci. 2020;21(12):4295. doi:10.3390/ijms21124295. 32560264 PMC7352642

[10] Meng L, Liu X, He C, Xu B, Li Y, Hu Y. Functional divergence and adaptive selection of KNOX gene family in plants. Open Life Sci. 2020;15(1):346–363. doi:10.1515/biol-2020-0036. 33817223 PMC7874613

[11] Hake S, Smith HM, Holtan H, Magnani E, Mele G, Ramirez J. THE ROLE OF KNOX GENES IN PLANT DEVELOPMENT. Annu Rev Cell Dev Biol. 2004;20:125–151. doi:10.1146/annurev.cellbio.20.031803.093824. 15473837

[12] Li D, Song Y, Zheng Y, Ma Y, Jiang X, Wang C, Li X. Research progress of class I KNOTTED-like homeobox genes in regulation of organ morphogenesis in plant. Plant Physiol Commun. 2020;56:1119–1126.

[13] Vollbrecht E, Veit B, Sinha N, Hake S. The developmental gene Knotted-1 is a member of a maize homeobox gene family. Nature. 1991;350:241–243. doi:10.1038/350241a0. 1672445

[14] Sinha NR, Williams RE, Hake S. Overexpression of the maize homeo box gene, KNOTTED-I, causes a switch from determinate to indeterminate cell fates. Genes Dev. 1993;7:787–795. doi:10.1101/gad.7.5.787. 7684007

[15] Ori N, Eshed Y, Chuck G, Bowman JL, Hake S. Mechanisms that control knox gene expression in the Arabidopsis shoot. Development. 2000;127(24):5523–5532. doi:10.1242/dev.127.24.5523. 11076771

[16] Zhang C, Wang R, Zhang Z. The habitat and distribution of *Lilium tsingtauense*. Hubei Agric Sci. 2010;49:1404–1406+1410.

[17] Guo W, Jeong J, Kim Z, Wang R, Kim Z, Kim S. Genetic diversity of *Lilium tsingtauense* in China and Korea revealed by ISSR markers and morphological characters. Biochem Syst Ecol. 2011;39:352–360. doi:10.1016/j.bse.2011.05.002.

[18] Murashige T, Skoog F. A revised medium for rapid growth and bio-assays with tobacco tissue culture. Physiol Plant. 1962;15:473–497. doi:10.1111/j.1399-3054.1962.tb08052.x.

[19] Kumar S, Stecher G, Li M, Knyaz C, Tamura K. MEGA X: molecular evolutionary genetics analysis across computing platforms. Mol Biol Evol. 2018;35(6):1547–1549. doi:10.1093/molbev/msy096. 29722887 PMC5967553

[20] Wang H, Li T, Li W, Wang W, Zhao H. Identification and analysis of Chrysanthemum nankingense NAC transcription factors and an expression analysis of OsNAC7 subfamily members. PeerJ. 2021;9:e11505. doi:10.7717/peerj.11505. 34123596 PMC8164415

[21] Fan M, Zhou R, Liu Q, Sun Y. CBF transcription factors involved in the cold response of Camellia japonica (Naidong). PeerJ. 2021;9:e12155. doi:10.7717/peerj.12155. 34589310 PMC8435204

[22] Zhu K, Shao M, Zhou D, Xing YX, Yang LT, Li YR. Functional analysis of Leifsonia xyli subsp. xyli membrane protein gene Lxx18460 (anti-sigma K). BMC Microbiol. 2019;19(1):2. doi:10.1186/s12866-018-1378-2. 30616519 PMC6323826

[23] Clarke JD. Cetyltrimethyl ammonium bromide (CTAB) DNA miniprep for plant DNA isolation. Cold Spring Harb Protoc. 2009;2009(3):db.prot5177. doi:10.1101/pdb.prot5177. 20147112

[24] Liu Z, Jia L, Mao Y, He Y. Classification and quantification of leaf curvature. J Exp Bot. 2010;61(10):2757–2767. doi:10.1093/jxb/erq111. 20400533 PMC2882270

[25] Sun X, Ma Y, Yang C, Li J. Rice OVATE family protein 6 regulates leaf angle by modulating secondary cell wall biosynthesis. Plant Mol Biol. 2020;104(3):249–261. doi:10.1007/s11103-020-01039-2. 32715397

[26] Zhang L, Li X. Comparative study of specimen preparation methods of malus xiaojinensis fruit epidermis. Acta Bot Boreal -Occcident Sin. 2013;33:2346–2350.

[27] Schneider CA, Rasband WS, Eliceiri KW. NIH Image to ImageJ: 25 years of image analysis. Nat Methods. 2012;9(7):671–675. doi:10.1038/nmeth.2089. 22930834 PMC5554542

[28] Bustin SA. Absolute quantification of mRNA using real-time reverse transcription polymerase chain reaction assays. J Mol Endocrinol. 2000;25(2):169–193. doi:10.1677/jme.0.0250169. 11013345

[29] Green MR, Sambrook J. Analysis and normalization of real-time polymerase chain reaction (PCR) experimental data. Cold Spring Harb Protoc. 2018;10:436–456. doi:10.1101/pdb.top095000. 30275081

[30] Jiang X, Chi X, Zhou R, Li Y, Li W, Liu Q, Wang K, Liu Q. Transcriptome profiling to identify tepal cell enlargement and pigmentation genes and the function of *LtEXLB1* in *Lilium tsingtauense*. Funct Plant Biol. 2020;48(3):241–256. doi:10.1071/FP20253. 33059816

[31] Zhang Y, Yong YB, Wang Q, Lu YM. Physiological and molecular changes during lily underground stem axillary bulbils formation. Russian J Plant Physiol. 2018;65(3):372–383. doi:10.1134/S1021443718030172.

[32] Jia P, Xing L, Zhang C, Chen H, Li Y, Zhang D, Ma J, Zhao C, Han M, Ren X, et al. MdKNOX15, a class I knotted-like transcription factor of apple, controls flowering and plant height by regulating GA levels through promoting the MdGA2ox7 transcription. Environ Exp Bot. 2021;185:104411. doi:10.1016/j.envexpbot.2021.104411.

[33] Zhang S, Pan Y, Zhi C, Zheng Y, Wang X, Li X, Cheng Z. Genome-wide identification and characterization of KNOTTED-like homeobox (KNOX) homologs in garlic (Allium sativum L.) and their expression profilings responding to exogenous cytokinin and gibberellin. Int J Mol Sci. 2021;22(17):9237. doi:10.3390/ijms22179237. 34502163 PMC8430937

[34] Kuijt SJ, Lamers GE, Rueb S, Scarpella E, Ouwerkerk PB, Spaink HP, Meijer AH. Different subcellular localization and trafficking properties of KNOX class 1 homeodomain proteins from rice. Plant Mol Biol. 2004;55(6):781–796. doi:10.1007/s11103-004-1967-3. 15604716

[35] Hackbusch J, Richter K, Müller J, Salamini F, Uhrig JF. A central role of Arabidopsis thaliana ovate family proteins in networking and subcellular localization of 3-aa loop extension homeodomain proteins. Proc Natl Acad Sci U S A. 2005;102(13):4908–4912. doi:10.1073/pnas.0501181102. 15781858 PMC555730

[36] Kim JY, Yuan Z, Jackson D. Developmental regulation and significance of KNOX protein trafficking in Arabidopsis. Development. 2003;130(18):4351–4362. doi:10.1242/dev.00618. 12900451

[37] Rim Y, Jung JH, Chu H, Cho WK, Kim SW, Hong JC, Jackson D, Datla R, Kim JY. A non-cell-autonomous mechanism for the control of plant architecture and epidermal differentiation involves intercellular trafficking of BREVIPEDICELLUS protein. Funct Plant Biol. 2009;36(3):280–289. doi:10.1071/FP08243. 32688646

[38] Srinivasan C, Liu Z, Scorza R. Ectopic expression of class 1 KNOX genes induce adventitious shoot regeneration and alter growth and development of tobacco (Nicotiana tabacum L) and European plum (Prunus domestica L). Plant Cell Rep. 2011;30(4):655–664. doi:10.1007/s00299-010-0993-7. 21212958

[39] Ikezaki M, Kojima M, Sakakibara H, Kojima S, Ueno Y, Machida C, Machida Y. Genetic networks regulated by ASYMMETRIC LEAVES1 (AS1) and AS2 in leaf development in Arabidopsis thaliana: KNOX genes control five morphological events. Plant J. 2010;61(1):70–82. doi:10.1111/j.1365-313X.2009.04033.x. 19891706

[40] Luo M, Yu CW, Chen FF, Zhao L, Tian G, Liu X, Cui Y, Yang JY, Wu K. Histone deacetylase HDA6 is functionally associated with AS1 in repression of KNOX genes in Arabidopsis. PLoS Genet. 2012;8(12):e1003114. doi:10.1371/journal.pgen.1003114. 23271976 PMC3521718

[41] Sentoku N, Sato Y, Matsuoka M. Overexpression of rice OSH genes induces ectopic shoots on leaf sheaths of transgenic rice plants. Dev Biol. 2000;220:358–364. doi:10.1006/dbio.2000.9. 10753522

[42] Frugis G, Giannino D, Mele G, Nicolodi C, Chiappetta A, Bitonti MB, Innocenti AM, Dewitte W, Van Onckelen H, Mariotti D. Overexpression of KNAT1 in lettuce shifts leaf determinate growth to a shoot-like indeterminate growth associated with an accumulation of isopentenyl-type cytokinins. Plant Physiol. 2001;126(4):1370–1380. doi:10.1104/pp.126.4.1370. 11500537 PMC117138

[43] Xu Q, Dong J, Gao N, Ruan M, Jia H, Zhang L, Wang C. Transgenic lines of Begonia maculata generated by ectopic expression of PttKN1. Biologia. 2011;66:251–257. doi:10.2478/s11756-011-0008-3.44.

[44] Lin L, Zhong SH, Cui XF, Li J, He ZH. Characterization of temperature-sensitive mutants reveals a role for receptor-like kinase SCRAMBLED/STRUBBELIG in coordinating cell proliferation and differentiation during Arabidopsis leaf development. Plant Journal. 2012;72(5):707–720. doi:10.1111/j.1365-313X.2012.05109.x. 22805005

[45] Liebsch D, Sunaryo W, Holmlund M, Norberg M, Zhang J, Hall HC, Helizon H, Jin X, Helariutta Y, Nilsson O, et al. Class I KNOX transcription factors promote differentiation of cambial derivatives into xylem fibers in the Arabidopsis hypocotyl. Development. 2014;141(22):4311–4319. doi:10.1242/dev.111369. 25371365

[46] Schneeberger RG, Becraft PW, Hake S, Freeling M. Ectopic expression of the knox homeo box gene rough sheath1 alters cell fate in the maize leaf. Genes Dev. 1995;9(18):2292–2304. doi:10.1101/gad.9.18.2292. 7557382

[47] Nishimura A, Tamaoki M, Sakamoto T, Matsuoka M. Over-expression of tobacco knotted1-type class1 homeobox genes alters various leaf morphology. Plant Cell Physiol. 2000;41(5):583–590. doi:10.1093/pcp/41.5.583. 10929941

[48] Horigome A, Nagasawa N, Ikeda K, Ito M, Itoh J, Nagato Y. Rice open beak is a negative regulator of class 1 knox genes and a positive regulator of class B floral homeotic gene. Plant Journal. 2009;58(5):724–736. doi:10.1111/j.1365-313X.2009.03823.x. 19207212

[49] Yan F, Hu G, Ren Z, Deng W, Li Z. Ectopic expression a tomato KNOX Gene Tkn4 affects the formation and the differentiation of meristems and vasculature. Plant Mol Biol. 2015;89(6):589–605. doi:10.1007/s11103-015-0387-x. 26456092

[50] Mahajan AS, Kondhare KR, Rajabhoj MP, Kumar A, Ghate T, Ravindran N, Habib F, Siddappa S, Banerjee AK. Regulation, overexpression, and target gene identification of Potato Homeobox 15 (POTH15) - a class-I KNOX gene in potato. J Exp Bot. 2016;67(14):4255–4272. doi:10.1093/jxb/erw205. 27217546 PMC5301930

[51] Li Z, Li B, Liu J, Guo Z, Liu Y, Li Y, Shen WH, Huang Y, Huang H, Zhang Y, et al. Transcription factors AS1 and AS2 interact with LHP1 to repress KNOX genes in *Arabidopsis*. J Integr Plant Biol. 2016;58(12):959–970. doi:10.1111/jipb.12485. 27273574

[52] Li Z, Li B, Shen WH, Huang H, Dong A. TCP transcription factors interact with AS2 in the repression of class-I KNOX genes in *Arabidopsis thaliana*. Plant J. 2017;71(1):99–107. doi:10.1111/j.1365-313X.2012.04973.x. 22380849

[53] Hay A, Barkoulas M, Tsiantis M. ASYMMETRIC LEAVES1 and auxin activities converge to repress BREVIPEDICELLUS expression and promote leaf development in Arabidopsis. Development. 2006;133(20):3955–3961. doi:10.1242/dev.02545. 16971475

[54] Sakamoto T, Kamiya N, Ueguchi-Tanaka M, Iwahori S, Matsuoka M. KNOX homeodomain protein directly suppresses the expression of a gibberellin biosynthetic gene in the tobacco shoot apical meristem. Genes Dev. 2001;15(5):581–590. doi:10.1101/gad.867901. 11238378 PMC312643

[55] Bolduc N, Hake S. The maize transcription factor KNOTTED1 directly regulates the gibberellin catabolism gene GA2ox1. Plant Cell. 2009;21(6):1647–1658. doi:10.1105/tpc.109.068221. 19567707 PMC2714931

[56] Rosin FM, Hart JK, Horner HT, Davies PJ, Hannapel DJ. Overexpression of a knotted-like homeobox gene of potato alters vegetative development by decreasing gibberellin accumulation. Plant Physiol. 2003;132(1):106–117. doi:10.1104/pp.102.015560. 12746517 PMC166957

[57] Nakayama H, Nakayama N, Seiki S, Kojima M, Sakakibara H, Sinha N, Kimura S. Regulation of the KNOX-GA gene module induces heterophyllic alteration in North American lake cress. Plant Cell. 2014;26(12):4733–4748. doi:10.1105/tpc.114.130229. 25516600 PMC4311196

[58] Sato Y, Sentoku N, Miura Y, Hirochika H, Kitano H, Matsuoka M. Loss-of-function mutations in the rice homeobox gene OSH15 affect the architecture of internodes resulting in dwarf plants. EMBO J. 1999;18:992–1002. doi:10.1093/emboj/18.4.992. 10022841 PMC1171191

[59] McHale NA, Koning RE. PHANTASTICA regulates development of the adaxial mesophyll in Nicotiana leaves. Plant Cell. 2004;16(5):1251–1262. doi:10.1105/tpc.019307. 15084717 PMC423213

[60] Tsuda K, Kurata N, Ohyanagi H, Hake S. Genome-wide study of KNOX regulatory network reveals brassinosteroid catabolic genes important for shoot meristem function in rice. Plant Cell. 2014;26(9):3488–3500. doi:10.1105/tpc.114.129122. 25194027 PMC4213158

[61] Barton MK, Poethig RS. Formation and maintenance of the shoot apical meristem in *Arabidopsis thaliana*: an analysis of development in the wild-type and in the shoot meristemless mutant. Development. 1993;119:823–831. doi:10.1242/dev.119.3.823.

[62] Long JA, Moan EI, Medford JI, Barton MK. A member of the KNOTTED class of homeodomain proteins encoded by the STM gene of Arabidopsis. Nature. 1996;379(6560):66–69. doi:10.1038/379066a0. 8538741

