# Supplementary material for: Overexpression of LtKNOX1 from Lilium tsingtauense in Nicotiana benthamiana affects the development of leaf morphology
Source: Plant Signal Behav. 2022 Feb 10;17(1):2031783. doi: 10.1080/15592324.2022.2031783 (PMC9176240; doi:10.1080/15592324.2022.2031783)
Supplement: Supplemental Material [file KPSB_A_2031783_SM9287.zip › Supplemental Figure S2.docx]

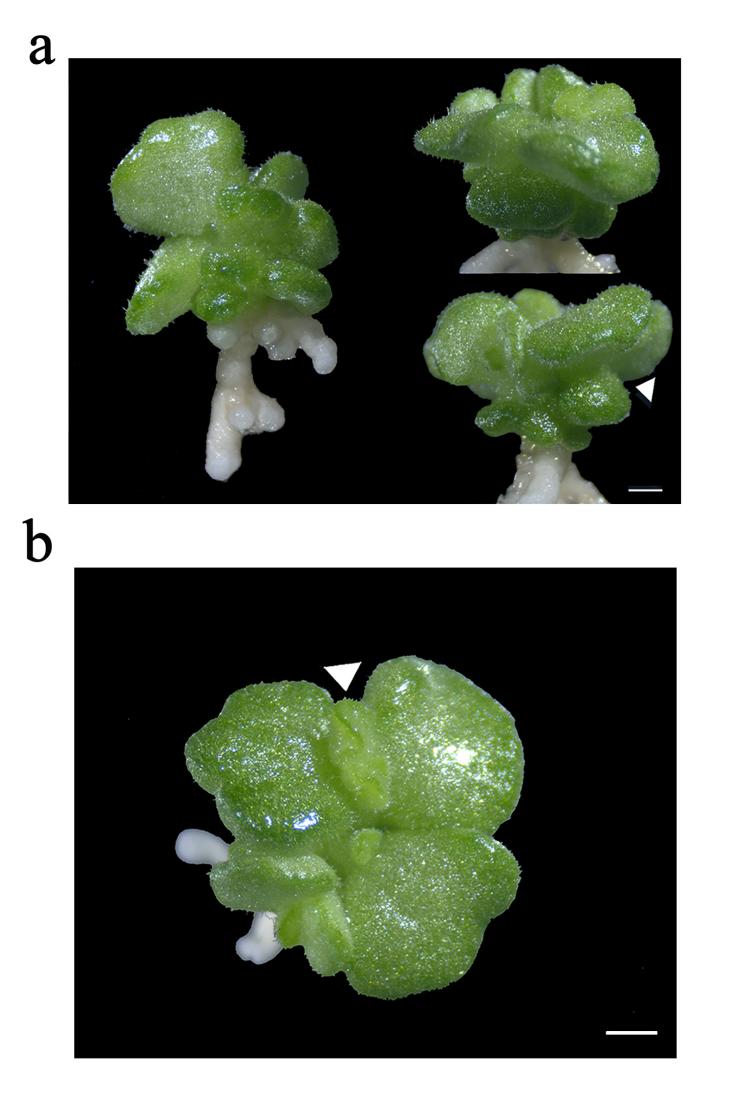


Figure S2. The phenotype of 50 DAG OE#2 observed by a stereomicroscope. White triangular arrows indicate developing leafy green organs. Scale bars=500 μm.
