# Supplementary material for: Overexpression of LtKNOX1 from Lilium tsingtauense in Nicotiana benthamiana affects the development of leaf morphology
Source: Plant Signal Behav. 2022 Feb 10;17(1):2031783. doi: 10.1080/15592324.2022.2031783 (PMC9176240; doi:10.1080/15592324.2022.2031783)
Supplement: Supplemental Material [file KPSB_A_2031783_SM9287.zip › Supplemental Table S1.docx]

Table S1. The sequence of *LtKNOX1*.

>*LtKNOX1* CDS

ATGGATGGCTTCACCCATCTCAGTGGCGGTGCCACCACCAGAGGTGGCGGCTTCATCTACTCCCCACCACCCCTTCACCTCACACCTTCAACCAGCTCATCCCTATACGACCGGACAATCTACTTTCCTCACCCACACAACCCTAACCCTAATCACCATGACCACATCAAACCAGAAGCAGGCTCTTCACAGGACACCGATGTGGAGTCCGTCAAGGCTCGGATCGTGGCCCACCCCCACTACTCCACCTTGCTTGCCGCCTACATGGAGTGCCAGAAGATCGGCGCCCCGCCGGAGGCGGCAGCCCGTCTCTCTGCTGCTGCCCGCGAGATGGAAGCCAGGCAGCGTGCCTCTGCGGAGAGTTCTCACATCGGCACCTCGACCGACCCGGAGTTGGATCAGTTCATGGAGGCATACTGCGATATGCTGGTAAAGTACAGGGAGGAGCTGACGAGGCCACTACAGGAGGCGATGGATTTCATGAAGAGGGTCGAATCGCAGCTCAACTCTTTGTGCTCTGATGGTGTTTCACCTCGCATCGTCTTCCCCACTGATGATAAATATGAAGGTGTTGGGTCATCGGAAGAGGACCAAGATGGCAGTGGGGGAGAGGCTGAAGTCCCTGAAATTGACCCGCGTGCCGAAGACAAAGAGCTAAAACACCACCTTCTGAAGAAGTACAGTGGATACTTGAGCAGCCTCAGACACGAACTCTCCAAGAAGAAGAAGAAAGGCAATCTCCCAAAGGATGCACGACAGAAGTTACTTAACTGGTGGGAGTTGCACTACAAATGGCCTTATCCATCGGAGACGGAGAAGGTAGCATTGGCAGAATCGACGGGCCTCGATCAGAAGCAGATCAACAATTGGTTCATAAACCAGAGGAAGCGGCACTGGAAGCCTTCAGAGGACATGAGGTTTGTTGTCATGGATGGCTTTCATCCTCAGAATGCTGCTGCTCTTACATGA

>LtKNOX1

MDGFTHLSGGATTRGGGFIYSPPPLHLTPSTSSSLYDRTIYFPHPHNPNPNHHDHIKPEAGSSQDTDVESVKARIVAHPHYSTLLAAYMECQKIGAPPEAAARLSAAAREMEARQRASAESSHIGTSTDPELDQFMEAYCDMLVKYREELTRPLQEAMDFMKRVESQLNSLCSDGVSPRIVFPTDDKYEGVGSSEEDQDGSGGEAEVPEIDPRAEDKELKHHLLKKYSGYLSSLRHELSKKKKKGNLPKDARQKLLNWWELHYKWPYPSETEKVALAESTGLDQKQINNWFINQRKRHWKPSEDMRFVVMDGFHPQNAAALT
