# Supplementary material for: Overexpression of LtKNOX1 from Lilium tsingtauense in Nicotiana benthamiana affects the development of leaf morphology
Source: Plant Signal Behav. 2022 Feb 10;17(1):2031783. doi: 10.1080/15592324.2022.2031783 (PMC9176240; doi:10.1080/15592324.2022.2031783)
Supplement: Supplemental Material [file KPSB_A_2031783_SM9287.zip › Supplemental Table S2.docx]

Table S2. The primers used in the study.

| Primer used in constructing vectors | Primer-F (5’-3’) | Primer-R (5’-3’) |
| --- | --- | --- |
| pSUPER1300-  *LtKNOX1* | CCCAAGCTTACCCGCTTCTCCTTCCATTA | GACTAGTTGTGGCAACAATTATACTTAGAATT |
| pSUPER1300 GFP-*LtKNOX1* | CCCAAGCTTATGCATGTCTTCACCCATCTCACTG | GACTAGTCGGGCCCAGGCGGTATGTAGCA |
| pSUPER1300 |  | TACTTGTACAGCTCGTCCATGCC |

| Primer used in qRT-PCR | Primer-F (5’-3’) | Primer-R (5’-3’) |
| --- | --- | --- |
| *LtGAPDH* | ACAGAAATGGCGTCGGTTCT | CGCTAGATCCTGAGTCGGTG |
| *LtKNOX1* | CCATCGGAGACGGAGAAGGTA | AAGCCATCCGTGACAACAAAC |
| *NbGAPDH* | AGCTCAAGGGAATTCTCGATG | AACCTTAACCATGTCATCTCCC |
| *NbAS1* | GAAGATTGTGAAAGAGCGGAGTT | AGCCACGGAGGAAGAAGAGTT |
| *NbCLV1* | AATCCCGACTTCCATTGCTCT | GGCAGGATTCAGTTGGTTTCG |
| *NbCUC2* | CGGTGCTAAGTCAAATGGTGG | GAAGAATCAAGGAGCGGTGGA |
| *NbGA20ox* | ATAGGCGATACATTTATGGCTCTT | TGGGCTCACTACCTTATCCTTCT |
| *NbPIN1* | TGCCCTGCCATCTGATAGTGT | ACCAAGTGACGCCAATGATGC |
| *NbWUS* | CAGAACCATAAAGCCCGTGAA | GCCAGTATGCCGTGAGAAGAA |
