# Supplementary material for: Overexpression of LtKNOX1 from Lilium tsingtauense in Nicotiana benthamiana affects the development of leaf morphology
Source: Plant Signal Behav. 2022 Feb 10;17(1):2031783. doi: 10.1080/15592324.2022.2031783 (PMC9176240; doi:10.1080/15592324.2022.2031783)
Supplement: Supplemental Material [file KPSB_A_2031783_SM9287.zip › Supplemental Table S3.docx]

Supplemental Table 3. The accession numbers of the sequences used in the phylogenetic tree.

| Species | Gene | GenBank accession number |
| --- | --- | --- |
| *Arabidopsis thaliana* | *AtKNAT1* | AY080834.1 |
| *Arabidopsis thaliana* | *AtSTM* | U32344 |
| *Arabidopsis thaliana* | *AtKNAT2* | U14175 |
| *Arabidopsis thaliana* | *AtKNAT6* | NM_180620.2 |
| *Arabidopsis thaliana* | *AtKNAT3* | X92392 |
| *Arabidopsis thaliana* | *AtKNAT4* | NM_121144.3 |
| *Arabidopsis thaliana* | *AtKNAT5* | NM_119356.3 |
| *Arabidopsis thaliana* | *AtKNAT7* | AF308451 |
| *Asparagus asparagoides* | *AaKNAT1* | AB673047.1 |
| *Asparagus officinalis* | *AoKNAT1* | AB673048.1 |
| *Brassica rapa* | *BrKNOX1* | XM_009135833 |
| *Cardamine hirsuta* | *ChKNOX1* | DQ630764.1 |
| *Dendrobium catenatum* | *DcKNOX1* | XM_028699021.1 |
| *Elaeis guineensis* | *EgKNOX1* | XM_010945104.3 |
| *Gossypium hirsutum* | *GhKNOX1* | XM_016893500.2 |
| *Lilium tsingtauense* | *LtKNOX1* | OK554547 |
| *Lilium* ‘Aladdin’ (*Lilium longiflorum × Lilium asiatic*) | *LiKNOX1* | KY965067.1 |
| *Manihot esculenta* | *MeKNOX1* | XM_021755832.2 |
| *Oryza sativa* | *OSH1* | JQ2379 |
| *Raphanus sativus* | *RsKNOX1* | XP_018475927.1 |
| *Zea mays* | *ZmKN1* | AAP76321.1 |
